# Wearable multichannel-active pressurized pulse sensing platform

**DOI:** 10.1038/s41378-024-00703-7

**Published:** 2024-06-11

**Authors:** Yunlong Zhao, Qingxia Sun, Shixuan Mei, Libo Gao, Xikuan Zhang, Zekun Yang, Xueli Nan, Haiyan Zhang, Chenyang Xue, Junyang Li

**Affiliations:** 1 Pen-Tung Sah Institute of Micro-Nano Science and Technology, Xiamen University, 361102 Xiamen, China; 2 Discipline of Intelligent Instrument and Equipment, Xiamen University, 361102 Xiamen, China; 3 Department of Electronic Engineering, Ocean University of China, 266000 Qingdao, China; 4 School of Automation and Software Engineering, Shanxi University, 030006 Taiyuan, China; 5 Innovation Laboratory for Sciences and Technologies of Energy Materials of Fujian Province (IKKEM), 361005 Xiamen, China; 6 Key Laboratory of Instrumentation Science and Dynamic Measurement Ministry of Education, North University of China, 030051 Taiyuan, China; 7 Science and Technology on Vacuum Technology and Physics Laboratory, Lanzhou Institute of Physics, Chinese Academy of Space Technology, 730000 Lanzhou, China

**Keywords:** Electrical and electronic engineering

## Abstract

With the modernization of traditional Chinese medicine (TCM), creating devices to digitalize aspects of pulse diagnosis has proved to be challenging. The currently available pulse detection devices usually rely on external pressure devices, which are either bulky or poorly integrated, hindering their practical application. In this work, we propose an innovative wearable active pressure three-channel pulse monitoring device based on TCM pulse diagnosis methods. It combines a flexible pressure sensor array, flexible airbag array, active pressure control unit, advanced machine learning approach, and a companion mobile application for human–computer interaction. Due to the high sensitivity (460.1 kPa^−1^), high linearity (*R*^2^ > 0.999) and flexibility of the flexible pressure sensors, the device can accurately simulate finger pressure to collect pulse waves (Cun, Guan, and Chi) at different external pressures on the wrist. In addition, by measuring the change in pulse wave amplitude at different pressures, an individual’s blood pressure status can be successfully predicted. This enables truly wearable, actively pressurized, continuous wireless dynamic monitoring of wrist pulse health. The innovative and integrated design of this pulse monitoring platform could provide a new paradigm for digitizing aspects of TCM and other smart healthcare systems.

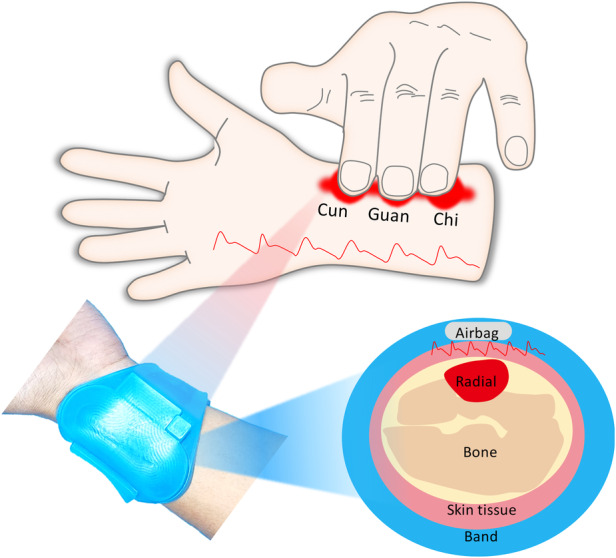

## Introduction

Traditional Chinese medicine (TCM) pulse diagnosis is a distinctive diagnostic approach that has demonstrated simplicity, effectiveness, and noninvasiveness in extensive clinical practice^1^. However, the process of TCM pulse diagnosis is heavily dependent upon the practitioner’s subjective sensations and practical experience, making it difficult to unify diagnostic standards^2^. This dependency significantly complicates the learning curve for novices in mastering pulse diagnosis techniques^3^. Such challenges have led to skepticism regarding the accuracy and objectivity of TCM pulse diagnosis in practical applications^4–7^. Integrating the abstract personal sensations of traditional pulse diagnosis with modern medical data platforms remains a significant challenge for the synergistic development of traditional pulse diagnosis and the modern medical system.

The process of pulse diagnosis can be digitized and instrumented through the application of sensors and automated control methods^8^. Pulse wave signal acquisition is the first step of digital pulse diagnosis research^9,10^. Various schemes have been proposed for digitally collecting pulse wave signals. Initial studies predominantly employed rigid sensing devices that were complex and bulky^11–14^. These sensing units cannot adhere closely to the skin, making the process susceptible to external interference and inconvenient for flexible pulse collection. With the development of flexible sensing technology^15–18^, researchers have turned to the use of flexible pressure sensing units^19–23^ or sensor arrays^24–26^ to replace traditional rigid sensors for detecting pulse waves from the skin surface. These flexible pressure sensors, characterized by their simple design and increased sensitivity^27–31^, have alleviated problems related to sensor-skin fit and environmental interference during data acquisition. However, most of the related research has concentrated on improving the efficiency of signal capture and developing innovative sensing mechanisms^32^. In addition, the wired connections for interfacing sensing units, signal processing, and display devices impede the development of a fully integrated system^33,34^.

Integrating sensitive sensors, signal acquisition, and processing circuits into a compact and portable design remains a significant challenge^35^. In particular, pulse diagnosis in traditional Chinese medicine can realize the perception of pulse wave information by changing the fingertip pressure and then changing the coupling degree between finger-sensitive neurons and the mechanical wave of the blood vessel wall. However, few studies have explored the integration of active pressure units into wearable pulse-sensing systems^36^.

In this study, we present a wearable pulse wave sensing platform with integrated active pressurization based on TCM pulse diagnosis methods. The system is designed as a wristband for human pulse monitoring and includes a mobile application for waveform display and data storage on a mobile phone. The system uses flexible pressure sensors, micro airbag pressurization, and an automatic pressurization control program that can simulate the process of TCM pulse-taking by compression and collects the wrist pulse waveforms from low to high pressure (0–50 kPa). The pressure sensing unit adopts an ion-sensitive material as the sensing layer, which has excellent sensitivity (460.1 kPa^−1^), linearity (*R*^2^ > 0.999) and stability (12,000 pressure cycles). Due to the digitization and stability of the entire platform, the acquisition error can be effectively reduced, improving the quantifiable and accurate results of each pulse diagnosis (pulse acquisition pressure and corresponding pulse waveform). In addition, compared with other wearable pulse wave sensing devices (Table S1), this work integrates a pulse sensing unit, pressure control unit, data processing unit and display unit, which solves the problem of active pressurization of wearable pulse sensing devices and allows the assessment of blood pressure (BP)-associated health conditions by changing the pulse wave amplitude from low to high pressure, which can help promote wearable pulse monitoring applications.

## Results and discussions

### Device fabrication and structural characterization

**Fig. 1:**
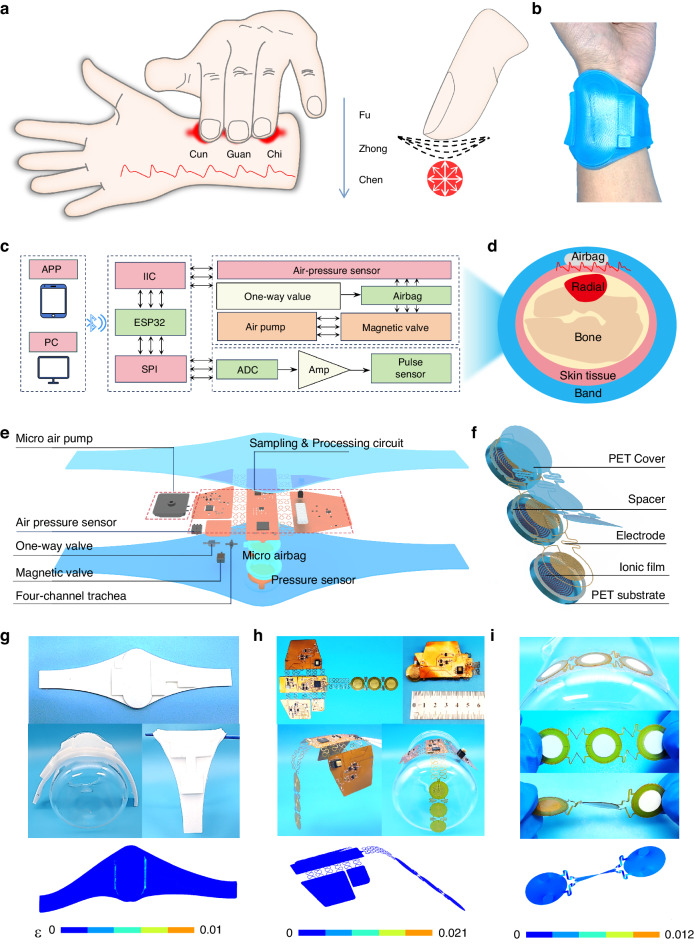
Overview and design of the flexible and wireless pulse sensing system. **a** Method of TCM pulse diagnosis. **b** Optical image of the wireless wristband worn on the user’s wrist joint. **c** Block functional diagram of the sensing system, including the power supply, signal acquisition, processing, communication, and user interface. **d** Schematic illustration of the wireless wristband worn on the wrist, where the airbag provides backpressure to effectively collect pulse wave changes under different pressures. **e** Detailed diagram of the overall structural design of the sensor system. **f** Detailed diagram of the overall structural design of the pressure sensor. **g**, **h**, and **i** Digital optical image and FEA results of the wristband, flexible circuit and sensing array under mechanical deformation

In TCM pulse diagnosis, it is believed that the health of human organs is related to the pressure pulse wave at corresponding mapping points (Cun, Guan, Chi) on the radial artery (Fig. 1a). In this study, we propose a wearable, flexible wristband that can be actively pressurized to mimic TCM pulse collection (Fig. 1b). The system comprises flexible pressure sensing units for collecting pulse waves at the “Cun, Guan, Chi” positions, an active pressure control unit providing different pressures, a wireless transmission unit for signal transmission and processing, a wireless charging unit for system power supply, and a power management unit.

The active pressure control unit, comprising silicone airbags, piezoelectric micropumps, a digital pressure sensor, electromagnetic valves, and one-way valves, works synergistically to provide precise pressure modulation. The micropump regulates airbag inflation, and pressure sensors and electromagnetic valves provide pressure feedback control (Fig. 1c, d). The hardware and software architecture of the system, including sensor integration, data processing modules, and user interface components, is comprehensively depicted in Fig. 1c. All components, such as the sensor array, micro-airbag array, micropump, and flexible printed circuit board (FPCB) and their interconnections, are encapsulated in soft silicone to create a fully flexible, wearable, multichannel active pressure pulse-sensing platform (WAPPP). This design allows the device to flex and stretch, ensuring tight and soft contact between the sensors and the arterial regions of the skin (Fig. 1d).

Figure 1e shows the hardware and software architecture of the system, including sensor integration, data processing modules, and user interface components. As shown in Fig. 1f, a 3-channel pulse sensor array was used to simulate three fingers for pulse wave acquisition. The overall structure of the pressure sensor includes three independent circular interdigital electrode resistance sensors, each with a diameter of 8 mm, which is slightly larger than the fingertip area of the human finger (Fig. S1). The three sensor units are connected by serpentine wires, significantly improving the deformability of the device and preventing mechanical interference between adjacent units. The pressurization of micro airbags ensures close contact between the sensor unit and the skin, enabling the precise conversion of local skin deformations caused by arterial expansion/contraction into electrical signal output. Figure 1g and h show that the system and its built-in flexible circuit board have excellent bending performance and can maintain good flexibility and equipment integration despite deformation. A flexible sensor array is easy to bend and mechanically stable. Figure 1i shows a digital optical image of the pressure sensor array and corresponding finite element analysis (FEA), demonstrating its applicability for wrist pulse measurements.

### Design and characterization of the pressure sensor

As a key part of pulse sensing systems, flexible pressure sensing arrays have high requirements for sensor performance. Resistance-type pressure sensors based on interdigital electrodes have advantages such as high sensitivity, high accuracy, high stability, convenient data collection, and simple device structures. In this paper, we used an interdigital electrode with a polyimide film (PI) substrate manufactured by FPCB technology as the induction electrode and thermoplastic polyether polyurethane (TPU)-ionic liquid (ILD)-h-BN as the ionic membrane.

**Fig. 2:**
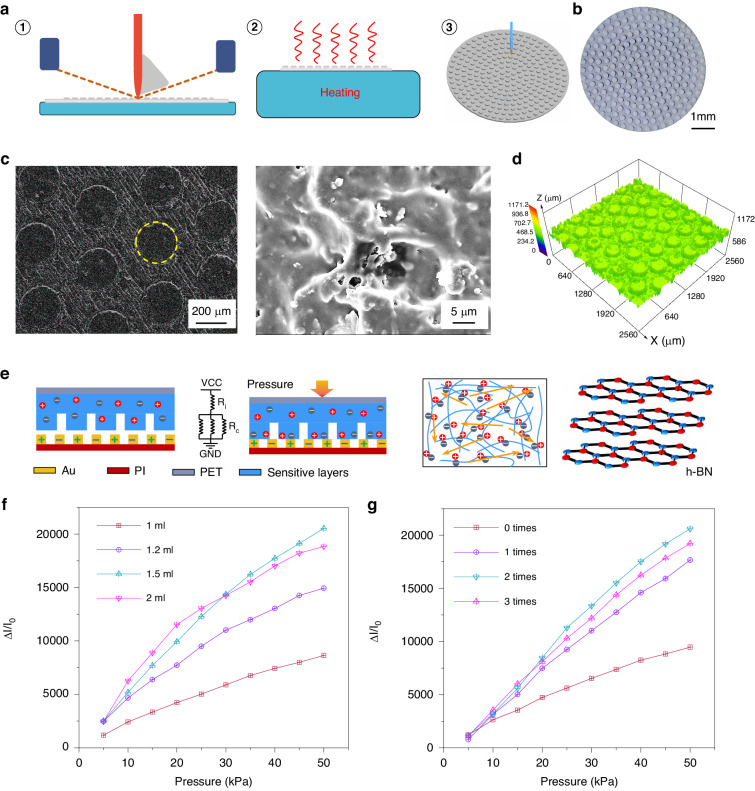
Pulse sensor design, fabrication and structural characterization. **a** Fabrication process of the pressure sensor. **b** Optical image of the sensitive layer with microstructure. **c** Illustration and scanning electron microscopy (SEM) images of the sensitive layer. **d** Sense LSCM image. **e** Schematic illustration and sensing mechanism of the pulse pressure sensor. **f** Current variation in sensors prepared with different ionic liquid contents. **g** Current variation in sensors prepared by different laser etching times

The sandwich structure is combined through bonding layers and a hot-pressing process to form an iontronic pressure sensor. The sensitive layer of the sensor was manufactured using screen printing, a process that is controllable in batches, as depicted in Figs. 2a and S2. After heat curing (Fig. 2a②), the sensitive layer was endowed with microcolumnar microstructures via laser engraving (illustrated in Fig. 2a**③**). The surface morphology of this layer is presented in microphotographs (Fig. 2b), scanning electron microscopy (SEM) maps (Fig. 2c), and laser scanning confocal microscopy (LSCM) images (Fig. 2d). These microcolumnar structures substantially enhance the deformation capability of the sensitive layer under compression, thereby significantly improving its sensitivity. Figure 2e shows the corresponding equivalent circuit, which indicates that the main variation in resistance within the circuit is due to the internal resistance (*R*_in_).

The doping of h-BN in the sensitive layer increased the viscosity of the printing paste and significantly improved the conductivity variation of the sensitive layer during deformation through the ion pump effect^27^. To explore the optimal performance of the sensor, we investigated the effect of different laser irradiation times (0, 1, 2, and 3) and various ionic concentrations (1, 1.2, 1.5 and 2 mL) on the sensor sensitivity. The results showed that the best performance for the sensitive layer was achieved with 1.5 mL of ionic liquid and 2 laser engravings. This was selected as the final sensor fabrication parameter (Fig. 2f, g).

**Fig. 3:**
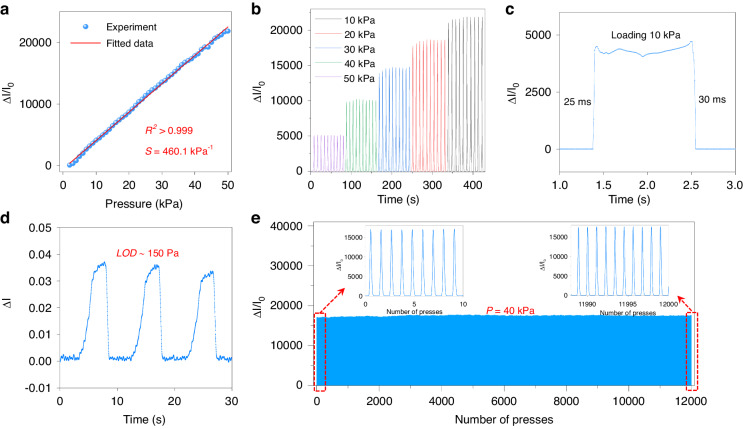
Sensor characterization. **a** Current variation versus pressure change of the pressure sensor. **b** Current variation of the pressure sensor under various pressures. **c** Fast response of the pressure sensor. **d** LOD of the sensor. **e** Long-term cycling ability of the sensor at 40 kPa for 12,000 cycles

To provide further evidence of the performance of the sensor, we conducted a series of tests and measurements to characterize its electrical performance (Fig. S7). The pressure sensor exhibits high sensitivity and good linearity within the pressure range of 0–50 kPa. As a crucial parameter for sensors, sensitivity is defined as *S* = (Δ*I*/*I*_0_)/Δ*P*. Here, *I*_0_ and Δ*I* represent the initial current under a 1 V voltage before loading and the change in the output current when pressure is applied, respectively. Figure 3a shows that the sensitivity of the pressure sensor is *S* = 460.1 kPa^−1,^ and the fitting coefficient is *R*^2^ > 0.999. It is noteworthy that the performance of this sensor surpasses that of most reported pressure sensors, enabling its suitability for testing scenarios such as human pulses and BP. We tested a series of continuous pressures to evaluate the sensor’s performance in this context. The sensor exhibits excellent consistency and mechanical robustness in the pressure sensing range of 0–50 kPa, making it highly effective for real-world applications and enhancing its practical applicability (Fig. 3b). Figure 3c shows that the pressure sensor response time and recovery time are 25 and 30 ms, respectively, which meet the requirements for pulse monitoring applications. To demonstrate the good resolution of the sensor, we characterized the limit of detection (LOD) of the sensor, which produces a response of ~0.035 μA at a pressure of 150 Pa, further verifying that the LOD of the sensor is approximately 150 Pa (Fig. 3d). Furthermore, the sensor demonstrated high stability and durability in long-term (12,000 cycles) pressure loading‒unloading cycles at 40 kPa (Fig. 3e).

### Design and application of silicone airbags

**Fig. 4:**
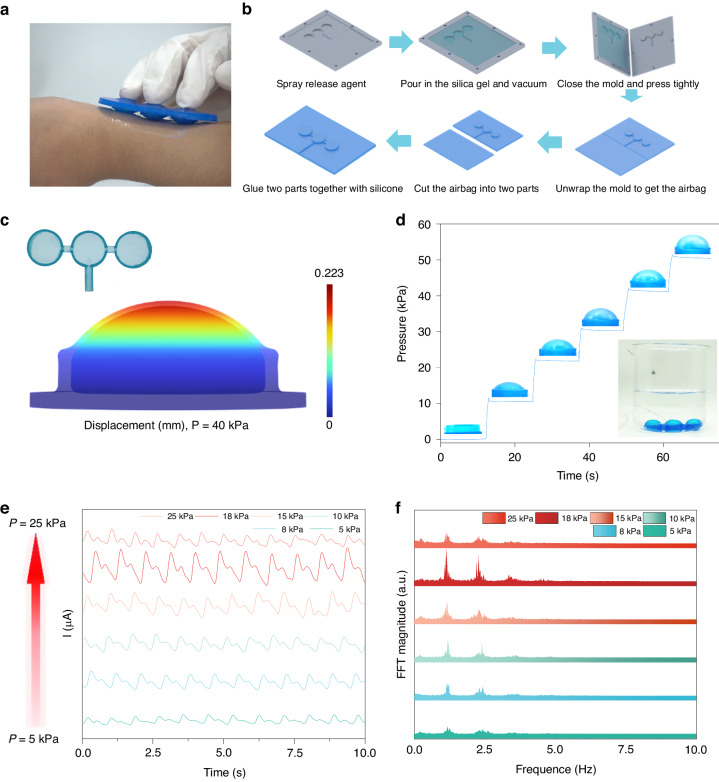
Characterizations of the airbag. **a** Digital optical image of the sensor patch on skin. **b** Fabrication process of the airbag. **c** Optical image of the airbag and stress‒strain simulation at 40 kPa. **d** The pressure inside the airbag is maintained within a stable range of 0–50 kPa. **e** and **f** With increasing pressure (5–25 kPa), the pulse amplitude and corresponding FFT results change

The active pressurization device comprises a micropump (19 mm × 21 mm × 3.6 mm, Fig. S4), a soft silicone (Ecoflex) airbag array and a one-way valve. Under pressure from the airbag array, the sensor array can detect mechanical pulses caused by the propagation of blood (Fig. 4a). Figure 4b shows the fabrication process of the micro airbag array. The piezoelectric micropump (Murata Machinery) controls the internal pressure of the silicone airbag and provides a controllable back pressure to the sensor array through conformal contact. FEA showed that the protruding displacement of the airbag surface was 2.223 mm when the pressure inside the airbag was 40 kPa, demonstrating the feasibility of using microairbags for the pressurized detection of pulse signals (Fig. 4c). The micropump supplies sufficient pressure (up to 50 kPa) to the airbag array, enabling steady pressure support for the sensor array (Supplemental Movie 1). The one-way valve at the outlet of the micropump serves as a pressure regulator to maintain pressure within the airbag while acting as a damping valve to stabilize the active pressure adaptive system. Figure 4d shows that the pressure in the airbag is basically unchanged when the air pump is used to inflate it to 10, 20, 30, 40, and 50 kPa at specific time intervals.

During actual pulse acquisition, with increasing external pressure, the coupling degree between the sensor and blood vessel changes. The amplitude of the pulse wave gradually increases and then decreases, as confirmed by the FFT results, which also demonstrate corresponding changes in frequency components with variations in the amplitude of the pulse wave (Fig. 4e).

### Applications for continuous pulse collection and BP estimation

**Fig. 5:**
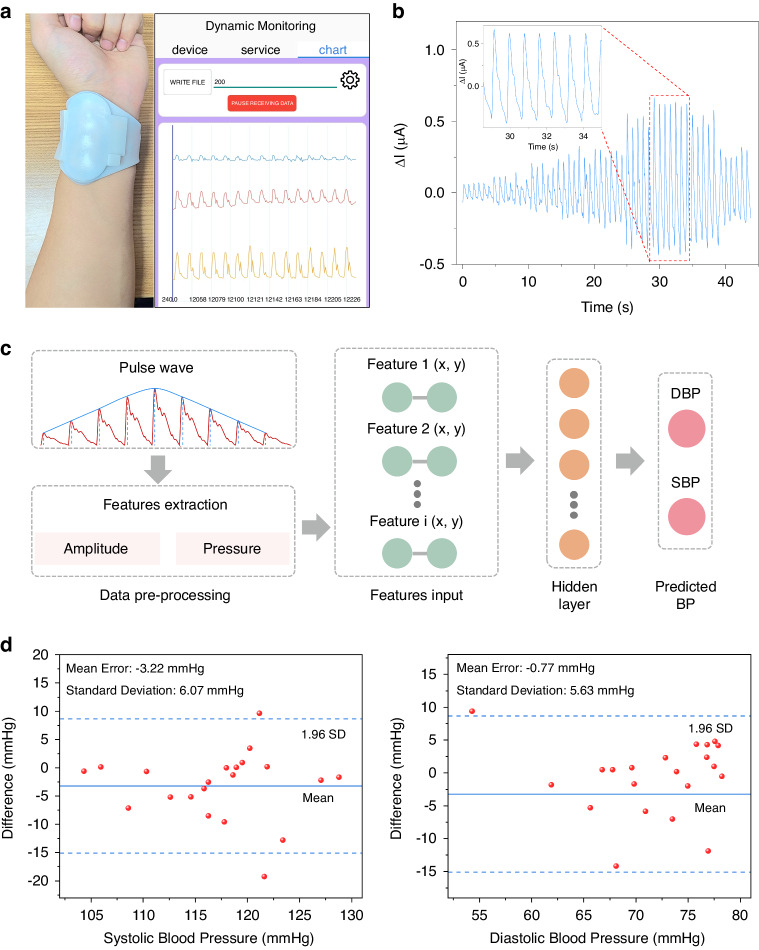
Practical application of the sensing system for monitoring pulse and blood pressure. **a** The display interface for mobile devices. **b** Pulse wave changes under 9 different static pressures. **c** BP prediction model. **d** Bland‒Altman plots to validate the accuracy of the pulse sensing system for SBP and DBP

The device can wirelessly connect to a compatible smartphone app via Bluetooth, enabling the transmission of pressure sensor signals to the mobile device for data storage and analysis (Fig. 5a and Supplement Movie 2). The WAPPP is based on controllable active airbag pressurization, which allows for the control of the sensor’s press depth, enabling the collection of pulse waves at different static pressures. The test results indicate that as the pressing force and depth increase, the amplitude of the pulse wave gradually increases, followed by a decrease (Fig. 5b), which is consistent with the theory of pulse diagnosis in TCM.

To validate the system’s applicability, we integrated a pulse wave test with a machine learning model and constructed a blood pressure prediction model based on a back-propagation neural network. This allows for accurate monitoring of blood pressure and cardiac status using the applied pressure and its corresponding pulse wave magnitude as input variables, inspired by the principle of blood pressure measurement. The back-propagation neural network was chosen for its flexible network structure and excellent nonlinear expression capabilities and is widely employed in BP prediction. In this study, we extracted pulse waveforms at nine pressure stages. After stabilizing the waveforms, we recorded the pulse amplitude values from the sensor and their corresponding airbag pressure values as inputs.

As illustrated in Fig. 5c, our approach utilizes a three-layer network structure comprising an input layer, a hidden layer, and an output layer. During model training, a single hidden layer is sufficient to fit high-precision functions. Using too many hidden layers can lead to overfitting and slow down the training process. The output layer consists of 2 nodes representing systolic and diastolic pressures. The pulse dataset is divided into three sets: training group, validation group, and testing group, with proportions of 70%, 15%, and 15%, respectively.

In the model training phase, as the back-propagation neural network receives data, it performs computations from the input layer through the hidden layer to the output layer, generating BP predictions. Through the adjustment of model parameters and correction with actual BP values, the corrected values are fed back into the input layer, enhancing the accuracy of the BP predictions. The results indicate a strong correlation (*R*-square value close to 0.99) between the output of the transfer function and that of commercial BP monitors (Fig. S5). Clinical validation of BP prediction was conducted using a test set of 21 BP data points. The average differences between our device and commercial BP monitors were −0.77 ± 9.0 mmHg for systolic blood pressure (SBP) and −3.22 ± 9.72 mmHg for diastolic blood pressure (DBP) (Fig. 5e, f). These BP prediction results met the American Association of Medical Instruments (AAMI) international criteria for BP testing.

By wearing the system on the user’s body, continuous and accurate monitoring of pulse variations can be achieved, allowing for the prediction of blood pressure. These results highlight the potential applications of the pulse acquisition system.

## Conclusions

The goal of an ideal pulse digitization acquisition process is to minimize manual intervention and increase automation. This paper focused on pulse signal acquisition and applied the theory of the TCM pulse diagnostic process to develop a flexible and integrated wearable active pressurized pulse sensing platform. The system integrates a flexible resistive pressure sensor (*S* = 460.1 kPa^−1^, 12,000 pressure cycles) and an active pressurized control unit (0–50 kPa). The system is portable and overcomes the limitations of using a separate flexible sensor unit without an integrated signal processing unit or active pressurization. The device can accurately detect and record pulse signals at multiple pressure gradients corresponding to three pulse locations (Cun, Guan, and Chi). The entire pulse collection process is controlled by a preset built-in program, which converts subjective sensations that cannot be quantified into digital signals, improving the automation level of pulse diagnosis and enabling real-time continuous visualization and collection of pulse wave data. This work addresses the issue of digitizing and standardizing the TCM pulse diagnosis data-gathering process. It is also significant in reducing subjective and individual differences in the process of Chinese medicine pulse diagnosis.

In addition, we extracted parameters related to the change in pulse amplitude as the external pressure gradually increased and constructed a back-propagation neural network-based blood pressure prediction model that combined the tested pulse data with a machine learning model to achieve accurate blood pressure prediction. The blood pressure prediction results met the AAMI international standard for blood pressure measurement. Predicting blood pressure using pulse waves also expands the availability of pulse data. We expect this work to play a key role in blood pressure monitoring, health monitoring, early diagnosis and telemedicine as sample data increases and data analysis strategies are optimized.

## Experimental design

### Flexible printed circuit board and communication

To ensure the ease of steady use, a thin FPCB was designed. The circuit’s structure adopts an island-bridge configuration, dividing the entire circuit into distinct functional modules based on their functions to reduce the circuit’s area. Snake-like conductive traces are employed to connect different functional modules, facilitating signal transmission among them. The entire system comprises a main control system, power system, air pump, and acquisition system. The entire circuit is fabricated using FPCB technology, allowing it to bend and adapt to the shape of the user’s arm for enhanced comfort during wear. The circuit’s power source involves a combination of batteries and wireless charging, enabling complete sealed packaging for improved dust and water resistance, thereby widening its range of applications.

### Design of signal processing and transmission circuits

All of the output signals from the sensors are collected by a microcontroller unit (MCU: ESP32-PICO-D4, STMicroelectronics) through an analog-to-digital converter (AD7799, Texas Instruments). A low-power Bluetooth module (DA14580, Dialog Semiconductor) facilitates data communication between the device and the PC (Figs. S6 and S7). The sampling rate of the pulse sensor is set at 50 Hz. The power source is a rechargeable lithium-ion battery with a capacity of 400 mAh. The device is equipped with a built-in wireless charging induction coil, allowing it to be charged by an external wireless charging device. Figure S8 shows the voltage and frequency of the wireless charging transmit coil and receiving coil (transmit: 12 V, receive: 6 V, frequency: 134 kHz), which meets the needs of the device’s operating voltage.

### Preparation of ion films with microcolumn structures

#### Preparation of the screen-printing slurry

2 g of transparent TPU particles were added to 5 mL of DMF solvent (Aladdin Company) and magnetically stirred at 120 °C (500 r/s) for 60 minutes until the TPU particles completely dissolved, forming a transparent liquid. Subsequently, 1.5 mL of an ionic liquid was added to the TPU solution, and the mixture was further heated and stirred at 120 °C for 60 min. Finally, 2 g of h-BN powder was added, and the mixture was magnetically stirred at 120 °C for an additional 60 min to obtain a pure white viscous printable slurry.

#### Preparation of the sensing layer

A transparent PET film with a thickness of 50 μm was selected as the substrate for the ion-sensitive layer and cut into small pieces of 10*15 cm^2^. The PET substrate surface underwent plasma cleaning to increase its hydrophilicity and roughness, thereby enhancing the adhesion between the printing paste and the PET substrate and improving the stability of the interfacial bond. Subsequently, using a 100-mesh screen, the ion-sensitive layer was screen-printed on a transparent PET substrate (50 μm) and cured completely by heating at 80 °C for 8–10 min. Then, a 250-mesh screen was used for the second printing on the cured ion-sensitive layer, followed by heating at 80 °C for 8–10 min to solidify. Finally, the microcolumns on the surface of the sensitive layer were engraved using a 355 nm UV laser (YLCF65U, Wuhan Yuanlu Co., Ltd.). The engraving process was repeated twice at a pulse frequency of 60 kHz and a pulse interval of 12 μs at a speed of 1000 mm s^−1^.

#### Sensor assembly

A circular 3M double-sided adhesive (with an outer diameter of 8 mm and an inner diameter of 7 mm) cut using a laser cutting machine was used for bonding the sensing layer and the interdigital electrode.

### Manufacture of airbags and silicone packages

The two components of the microairbags were manufactured by casting organic silicon elastomer (Ecoflex 00-30) with the assistance of a 3D printed mold. A specific quantity of silicone gel was weighed and mixed with a curing agent at a 100:2 ratio. The mixture was stirred thoroughly with a glass rod. Subsequently, the silicone gel was poured into the mold, and a vacuum was applied using a vacuum pump. Once the air bubbles disappeared entirely, the mold was removed and left to cure at room temperature for 5–6 h. After demolding, the silicone gel surface was coated with vacuum-extracted silicone gel, and the upper and lower parts, along with silicone tubing, were cured at room temperature for 5–6 h to achieve a stable interface connection. Finally, the outlet was sealed with silicone to facilitate connection with a one-way valve and a micropump. Silicone packaging was prepared in a similar way (Fig. S9).

### FEA of mechanics

FEA was carried out to study the mechanical behaviors of the silicone airbag and FPCB under diverse deformations, and all of the material properties were assigned according to the material sources in the FEA software. Young’s moduli of copper, Ecoflex, and polymide were set to 80 GPa, 60 kPa, and 800 MPa, respectively.

### Structural, mechanical, and electromechanical characterization

Structural and morphological information was obtained using a field emission scanning electron microscope (FESEM; Quanta 450, 20 kV). The mechanical properties were determined using a mechanical testing machine (Zhi Qu, 990B). Each test was repeated five times, and the average value was calculated to obtain the final result. For electromechanical characterization, the variable resistance, current, and capacitance during the process were recorded using an electrochemical workstation (CHI 760E) and an LCR impedance analyzer (IM3536 LCR meter).

### Ethical approval

All human subject studies were approved by the Medical Ethics Committee of Xia Men University (Protocol: XDYX202311K70), and the volunteers provided informed consent.

### Supplementary information

Supporting information for Publication

Supplement Movie1

Supplement Movie2
