# Supplementary material for: Wearable multichannel-active pressurized pulse sensing platform
Source: Microsyst Nanoeng. 2024 Jun 11;10:77. doi: 10.1038/s41378-024-00703-7 (PMC11166975; doi:10.1038/s41378-024-00703-7)
Supplement: Supplementary file 1 — Supporting information for Publication [file 41378_2024_703_MOESM1_ESM.docx]

**Wearable Multichannel Active Pressurized Pulse Sensing Platform**

Yunlong Zhao^1,2†^, Qingxia Sun^3†^, Shixuan Mei^4^, Libo Gao^1,2*^, Xikuan Zhang^5^, Zekun Yang^5^, Xueli Nan^4^, Haiyan Zhang^6*^, Chenyang Xue^1,2,7*^ and Junyang Li^3*^

^1^Pen-Tung Sah Institute of Micro-Nano Science and Technology, Xiamen University, Xiamen, 361102, China

^2^Discipline of Intelligent Instrument and Equipment, Xiamen University, Xiamen, 361102, China

^3^Department of Electronic Engineering, Ocean University of China, Qingdao 266000, China

^4^School of Automation and Software Engineering, Shanxi University, Taiyuan, 030006, China

^5^Key Laboratory of Instrumentation Science and Dynamic Measurement Ministry of Education, North University of China, Taiyuan, 030051, China

^6^Science and Technology on Vacuum Technology and Physics Laboratory, Lanzhou Institute of Physics, Chinese Academy of Space Technology, Lanzhou, 730000, China

^7^Innovation Laboratory for Sciences and Technologies of Energy Materials of Fujian Province (IKKEM), Xiamen, 361005, China

*Author to whom correspondence should be addressed:

E-mail: [lbgao@xmu.edu.cn](mailto:lbgao@xmu.edu.cn); zhanghy510@spacechina.com; xuechenyang@nuc.edu.cn; [lijunyang@ouc.edu.cn](mailto:lijunyang@ouc.edu.cn)

†These authors contributed equally to this work.

**Table S1 Comparison of different pulse sensing system**

| **Reference** | **Flexibility** | **Totally**  **wearable** | **Active pressure** | **Blood pressure** | **Wireless** | **Systemic**  **integration** |
| --- | --- | --- | --- | --- | --- | --- |
| 11 | 🗴 | 🗴 | 🗸 | 🗴 | 🗴 | 🗴 |
| 12 | 🗴 | 🗴 | 🗸 | 🗴 | 🗴 | 🗴 |
| 13 | 🗴 | 🗴 | 🗴 | 🗸 | 🗴 | 🗴 |
| 14 | 🗴 | 🗴 | 🗸 | 🗴 | 🗴 | 🗴 |
| 19 | 🗸 | 🗴 | 🗴 | 🗸 | 🗴 | 🗴 |
| 20 | 🗸 | 🗴 | 🗴 | 🗴 | 🗴 | 🗴 |
| 21 | 🗸 | 🗴 | 🗴 | 🗴 | 🗴 | 🗴 |
| 22 | 🗸 | 🗴 | 🗴 | 🗴 | 🗴 | 🗴 |
| 23 | 🗸 | 🗴 | 🗴 | 🗸 | 🗴 | 🗴 |
| 24 | 🗸 | 🗴 | 🗴 | 🗴 | 🗴 | 🗴 |
| 25 | 🗸 | 🗴 | 🗴 | 🗴 | 🗴 | 🗴 |
| This work | Full-soft | 🗸 | 🗸 | 🗸 | Bluetooth | 🗸 |


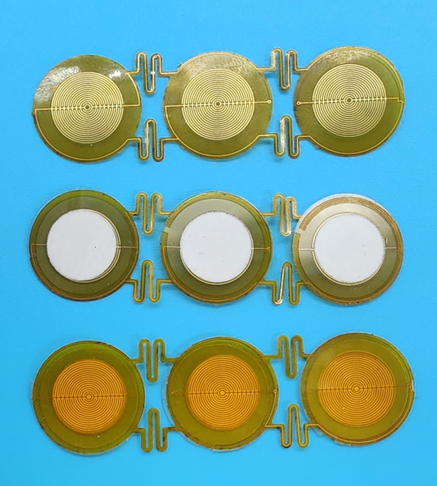
Figure S1. Optical diagram of the sensor. The optical diagram of the sensor includes a front image, a back image and an interdigital electrode image.


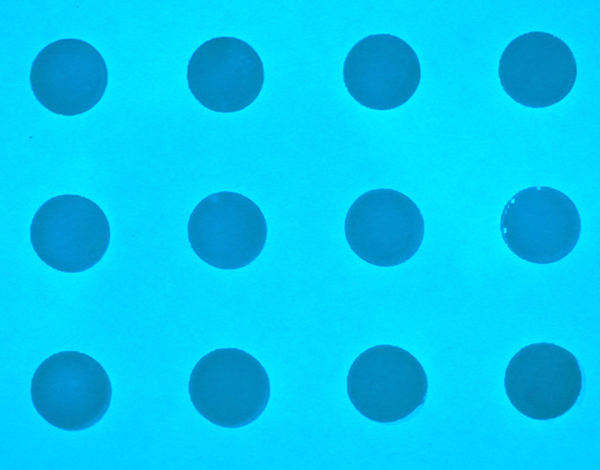
Figure S2. Optical diagram of the sensitive layer. The sensitive layer printed by screen printing technology.


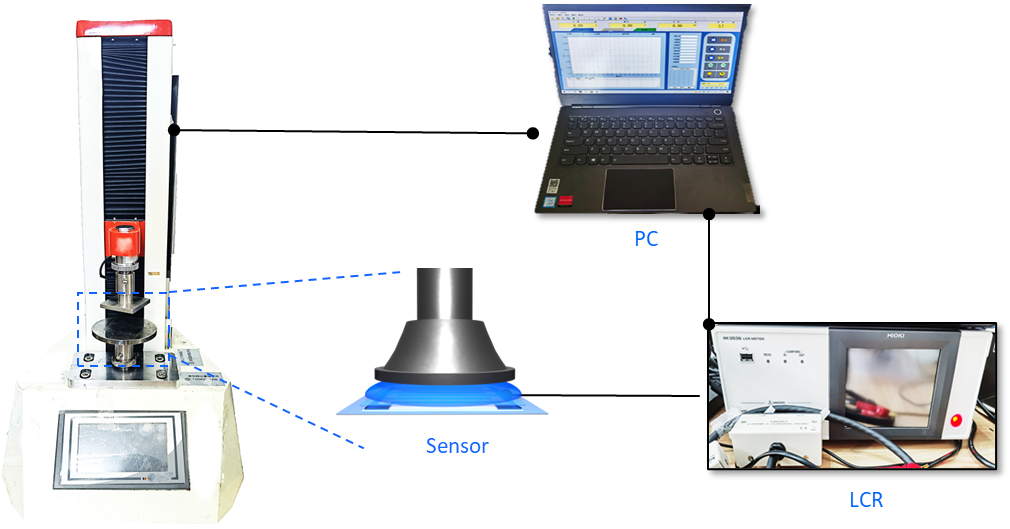


Figure S3. Experimental platform.


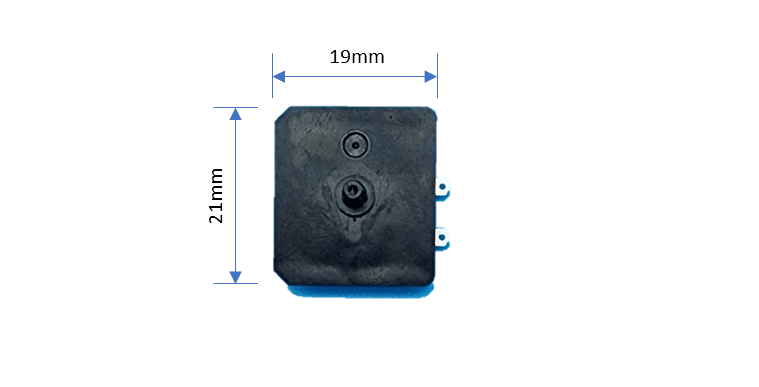


Figure S4. Dimensions of the miniature air pump.


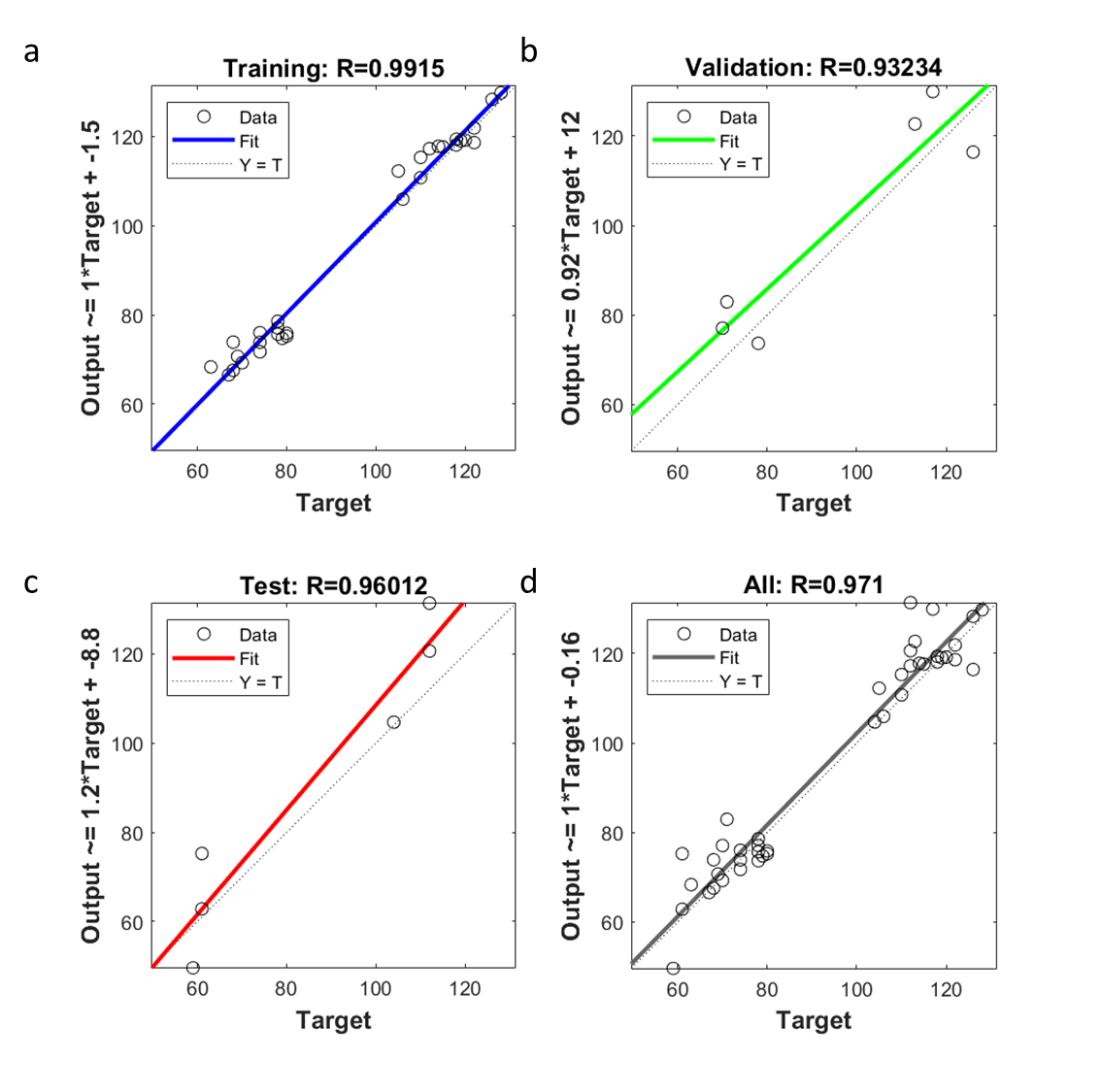


Figure S5. Fitting effect of BP neural network model performance. a Training set. b Verification set. c Test set. e Data set.


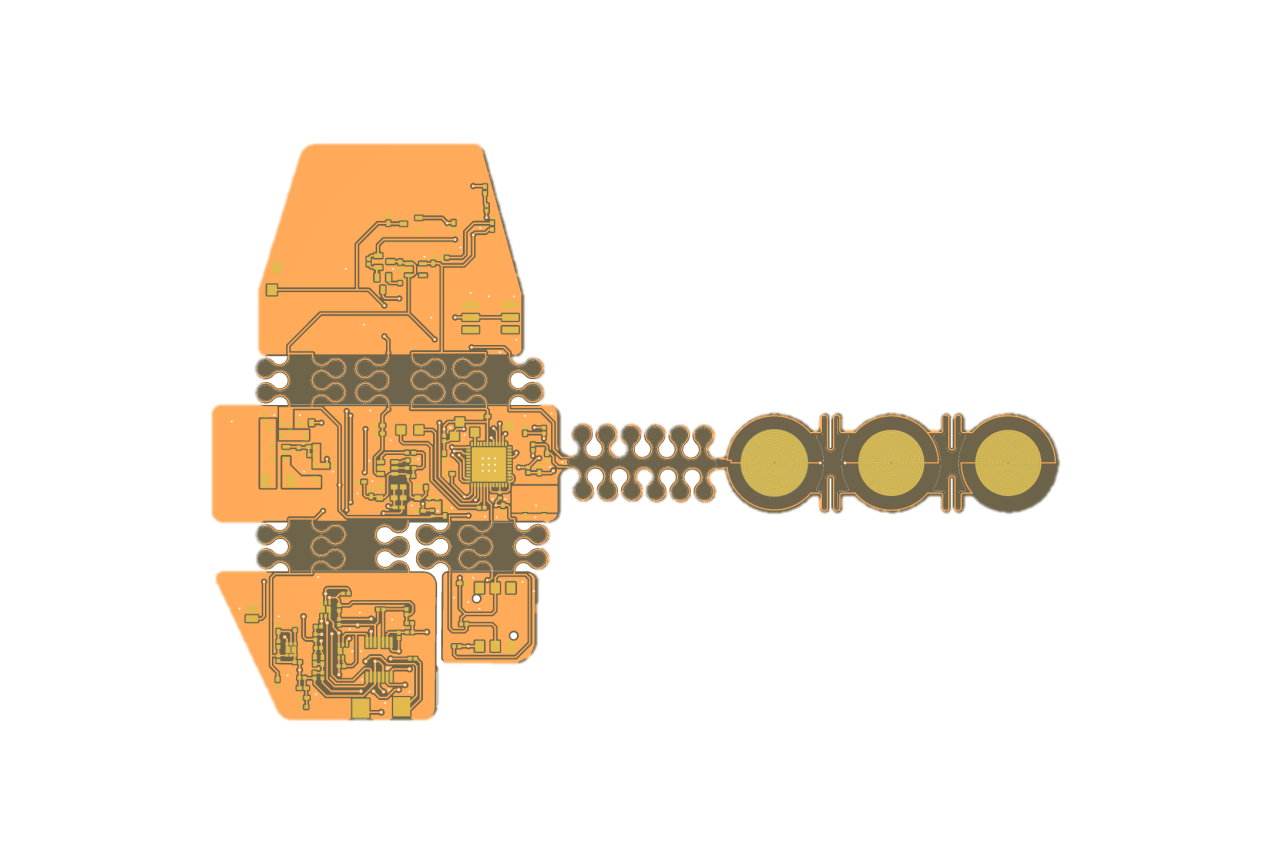
Figure S6. Circuit Design and Board Layout.


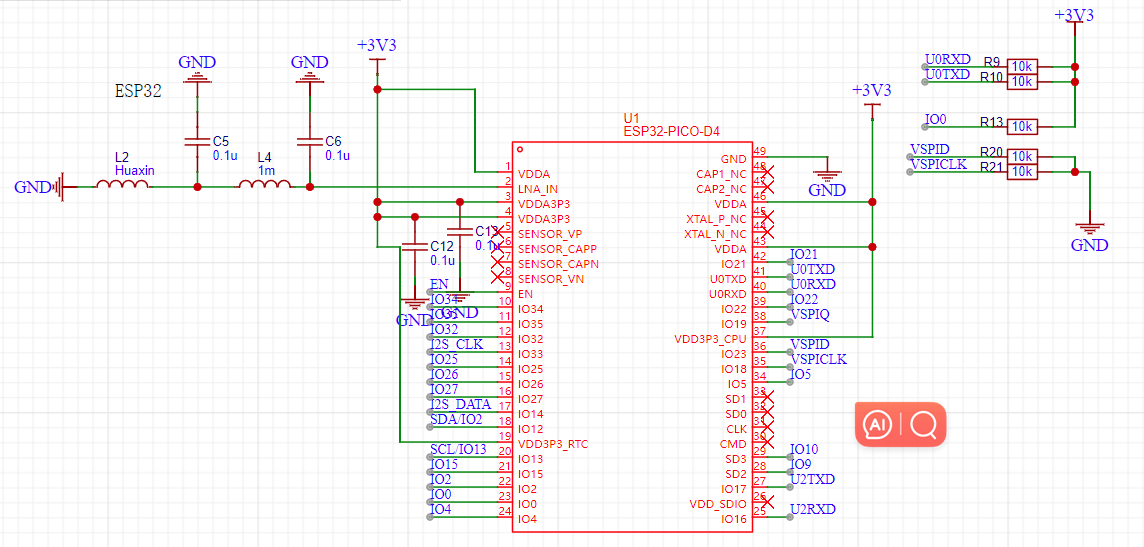


Figure S7 Schematic diagram of circuit.

**
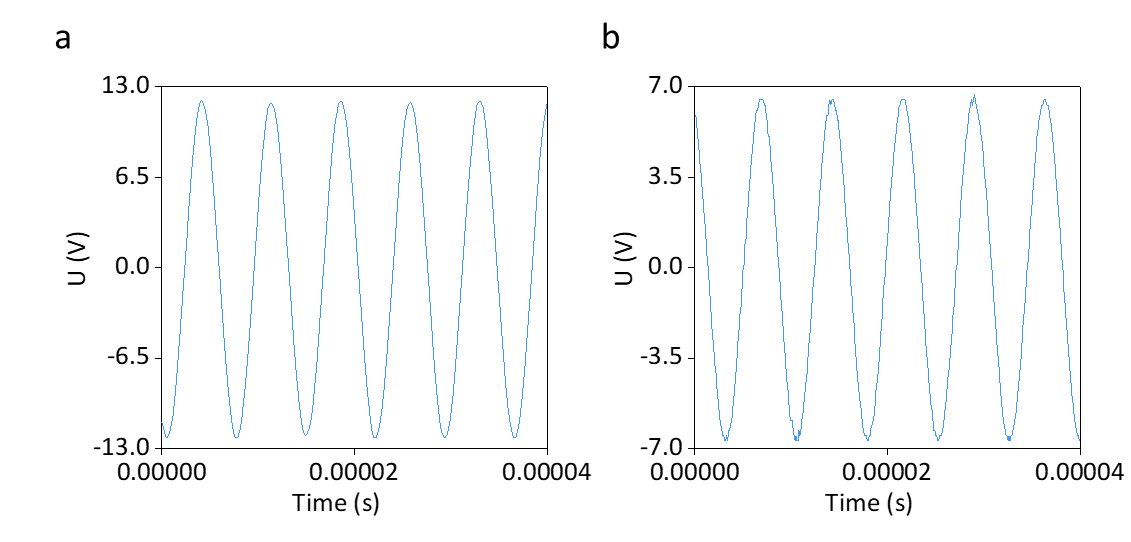
** Figure S8. Schematic diagram of each design module. a AC waveform diagram of wireless charging transmitting module. b AC waveform diagram of wireless charging receiving module.


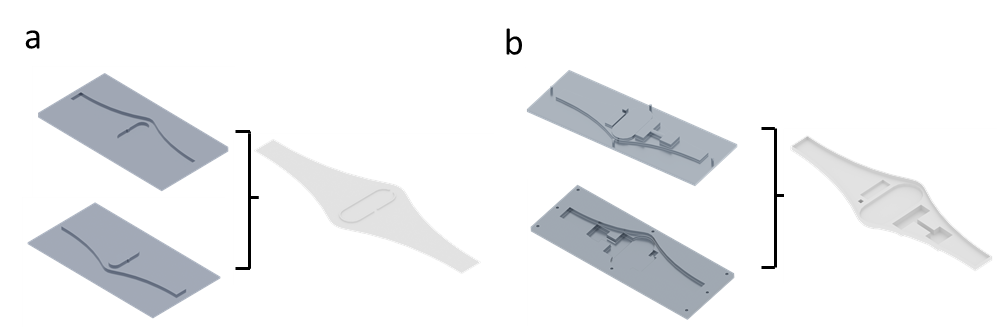
Figure S9. Production process of silicone shell. a Production process of the lower half silicone shell. b Production process of the upper half silicone shell.
